# Effects of *Antrodia camphorata* extracts on anti-oxidation, anti-mutagenesis and protection of DNA against hydroxyl radical damage

**DOI:** 10.1186/s12906-015-0768-3

**Published:** 2015-07-16

**Authors:** Yu-Lin Hsieh, Szu-Pei Wu, Li-Wen Fang, Tzann-Shun Hwang

**Affiliations:** Department of Nutrition, I-Shou University, No. 8, Yida Rd., Jiaosu Village, Yanchao District, Kaohsiung City, Taiwan; Department of Biotechnology and Pharmaceutical Technology, Yuanpei University, No. 306, Yuanpei St, Hsinchu City, Taiwan; Graduate Institute of Biotechnology, Chinese Culture University, No. 55, Hwa-Kan Rd, Yang-Ming-Shan, Taipei, Taiwan

**Keywords:** *Antrodia camphorata*, Submerged cultivation, Ames test, DNA damage, Antioxidant activity

## Abstract

**Background:**

*Antrodia camphorata* is a geographically special fungus and is one of the precious traditional medicines of Taiwan. A lot of reports have addressed its antioxidant activities and anticancer activities. In order to understand whether these protection effects were resulted from its ability of preventing DNA against hydroxyl radical damage, the *A. camphorata* extract was used to examine its antioxidant, antimutagenic and DNA-protective activities.

**Methods:**

*A. camphorata* extract was prepared by extracting the lyophilized powder of *A. camphorata* mycelium with distilled water. The antioxidative activity of this *A. camphorata* extract was then evaluated by 2,2-diphenyl-1-picrylhydrozyl (DPPH) radical-scavenging assay, and the antimutagenic activities of the extract against direct mutagen 4-nitroquinoline N-oxide (4NQNO) and indirect mutagen benzo[a]pyrene (B[a]P) were evaluated by Ames test. The effects of the *A. camphorata* extract in terms of DNA protection against hydroxyl radical damage were also investigated.

**Results:**

It was found that the higher the concentration of *A. camphorata* extracts, the higher the DPPH radical-scavenging effect. *A. camphorata* extract at concentrations between 0.625 and 10 mg/ml was found to be neither toxic nor mutagenic. However, the higher *A. camphorata* concentration (10 mg/ml) used in the test showed higher inhibitory effects on 4NQNO in a dose-dependent manner. The *A. camphorata* extract also showed reducing and scavenging activities against superoxide anion radical and also exhibited protective effects on DNA against hydroxyl radical-induced damage.

**Conclusions:**

Results suggested that *A. camphorata* is a non-toxic and novel material with antioxidant, antimutagenic and DNA-protective activities and could be developed into health foods.

## Background

*Antrodia camphorata* (also called Chang-Chih or Niu-Chang-Ku) is a member of the *Polyporaceae* (*Aphyllophorales*) family and hosts in brown heart rot of the endemic evergreen *Cinnamomum kanehirai* Hay (Lauraceae) in Taiwan [1]. The fruit body of *A. camphorata* is a well-known and expensive medicinal material in Taiwan. Traditionally, it is used as an antidote, anti-cancer, anti-itching and hepato-protective medicine. It has a mild camphor odor like that of its host tree, and the taste of it is very bitter. Currently, there is a shortage of the natural harvested fruit body of *A. camphorata* because its natural host, *C. kanehirai* Hay, is one of the endangered species in Taiwan.

Due to the rareness and growing slowly in natural environments, the fruit bodies of *A. camphorata* were expensive and difficult to procure; however, a breakthrough occurred in 1998 when a method of submerged culture for growing *A. camphorata* was established. Since the mycelium of *A. camphorata* was easy to obtain by submerged cultivation, many pharmacological effects of crude extracts of *A. camphorata* in different models of in vivo and in vitro studies were subsequently reported. These pharmacological effects were reviewed by Geethangili and Tzeng in 2009 [2] and classified into nine functional categories, including (1) Anti-cancer activities: Both the fruiting bodies and mycelium of *A. camphorata* was reported to have potent anti-proliferative activities against various cancers both in vitro and in vivo. (2) Anti-inflammatory and immunomodulatory effects. (3) Anti-hepatitis B virus replication: *A. camphorata* extracts was reported to have anti-hepatitis B virus activity in vivo in a dose-dependent manner without cytotoxicity. (4) Antioxidant activities: Accumulating data showed that *A. camphorata* could be a potent direct free radical scavenger. (5) Hepato-protective activity: *A. camphorata* was showed to have protective activity against liver hepatitis and fatty liver induced by acute hepatotoxicity of alcohol and also showed to have potential in treating liver diseases. (6) Prevention of liver fibrosis: The filtrate of fermented *A. camphorata* was found to have preventive and curative properties against rat liver fibrosis induced by CCl_4_-treatment. (7) Neuro-protective effects: Extracts from *A. camphorata* specimens grown via submerged cultivation were reported to prevent serum-deprived PC-12 cell apoptosis through a PKA-dependent pathway and by suppression of JNK and p38 activities. (8) Anti-hypertensive effects: Methanol extracts of *A. camphorata* showed potent antihypertensive effects in spontaneously hypertensive rats. (9) Vasorelaxation effects: The extracts of submerged cultural mycelium were reported to have vasorelaxation effects in a concentration-dependent manner.

At present, *A. camphorata* is mainly prepared via the submerged cultivation and used in the formulation of nutraceuticals and functional foods. It is of great interest to examine more functional properties of water extracts of *A. camphorata*. Few reports thus far, however, have been concerned with studies of the biological and chemical effects of *A. camphorata* extracts at the level of DNA function. Accordingly, the aim of this study was to elucidate the biological and chemical properties of *A. camphorata* extracts, including any effects such as mutagenic activity, anti-mutagenic activity against mutagens, and the protective effect of DNA from hydroxyl radical damage. The antioxidant activity and scavenging effects on radicals were also investigated in this study to compare with other reports.

## Methods

### *Antrodia camphorata* and its composition analyses

*A. camphorata* mycelium used in this study was a gift from Prof. Szu-Chuan Shen (National Taiwan Normal University, Taipei), which was produced by Simpson Biotech Co. Ltd. Water content and ash content of *A. camphorata* were determined according to the standard procedure by using the dried powder of *Antrodia camphorata* specimens grown by submerged cultivation. The total protein was determined by the Kjeldahl method, as described previously [3].

### Preparation of *A. camphorata* extract (ACE)

Dried *A. camphorata* mycelium from submerged cultivation was grounded into powder. The *A. camphorat*a extract (ACE) was prepared by water extraction. Two hundred milliliter of distilled water was added into 10 g of grounded powder, mixed well and stirred at 4 °C for 24 h. After centrifugation, the clear supernatant was collected, concentrated 10 times by rotary vapor machine, and lyophilized into dry powder by a lyophilyzer. The resultant dry powder was ACE and was stored at −20 °C for use in the following experiments.

### Evaluation of total phenolic compounds

The total phenoic content was determined by the following procedure and expressed in terms of milligrams of gallic acid equivalent per gram of aqueous extract [4]. The dried powder of ACE was dissolved in distilled water with a proper concentration. One hundred microlitre of ACE solution was added into 2.0 ml of 2 % Na_2_CO_3_, mixed well and held for 2 min. Folin-ciocalteu’s reagent (50 %) was then added into samples, mixed well and held for 30 min. The reacted samples were analyzed by spectrophotometer at 750 nm to determine the total phenolic compounds.

### Scavenging effect on 1,1-Diphenyl-2-picrylhydrazyl radicals

Scavenging ability on 1,1-Diphenyl-2-picrylhydrazyl (DPPH) radicals was measured by the method described previously [5]. Each water solution of ACE (0.5–10 mg/ml) was mixed with 1 ml of a methanolic solution containing DPPH (Sigma) radicals, resulting in a final concentration of 0.2 mM 1,1-diphenyl-2-picrylhydrazyl (DPPH). The mixture was shaken vigorously and left to stand for 30 min in the dark, and the absorbance was then measured at 517 nm. Antioxidant butylated hydroxytoluene (BHT) was used as the positive control. The scavenging activity (%) was defined as the percentage of the reduced absorbance caused by treating DPPH mixture with samples. The absorbance caused by DPPH mixture with water was used as the 100 % control.

$$ \mathrm{Scavenging}\ \mathrm{activity}\ \left(\%\right)=\frac{1-{\mathrm{A}}_{\mathrm{sample}}}{{\mathrm{A}}_{\mathrm{H}2\mathrm{O}}}\times 100\% $$

where A_sample_ is the absorbance caused by treating DPPH mixture with samples and A_H2O_ is the absorbance by treating DPPH mixture with water.

### Toxicity test

*Salmonella typhimurium* TA98 was obtained from the Bioresource Correction and Research Center (BCRC), FIRDI, Hsinchu, Taiwan. The strain was checked by examining its four genotypes according to the method described by Maron and Ames [6], including the histidine requirement in a biotin control plate and a biotin plate with histidine, rfa mutation test, uvr mutation test and R-factor confirmation.

If a sample has toxicity effects on *S. typhimurium* TA98 in an anti-mutagenic test, it will decrease the number of the testing bacteria and cause a wrong judgment for the data. In order to eliminate such potential error, a toxicity test of ACE was conducted. 0.1 ml of ACE was mixed with 0.5 ml of 0.2 M phosphate buffer (pH7.4) or S9 mixture, then 0.1 ml of activated *S. typhimurium* TA98 was added and incubated at 37 °C for 20 min. A 1 ml sample of the bacteria solution was aliquoted on to a plate, nutrient agar was added and the combination was mixed well. The colony formation units were counted after incubation at 37 °C for 48 h. Distilled water was used as control instead of sample. Colony count was monitored to check whether the colony number was reduced obviously; if yes, the concentration of ACE was further reduced until no toxicity was observed.

### Mutagenesis analysis

Mutagenesis analysis needs to be performed in addition of toxicity testing to eliminate possible errors due to a sample’s mutagenic effect, because if a sample has the ability to cause mutations, it will influence the number of revertants and result in an error in judgment. 0.1 ml of ACE was mixed with 0.5 ml of 0.2 M phosphate buffer (pH7.4) or S9 mixture, then 0.1 ml of activated *S. typhimurium* TA98 was added and incubated at 37 °C for 20 min. Then 2 ml of molten top agar (about 45 °C) was added into the tube, mixed well and then poured into a minimal glucose agar plate. Colonies in the plate were counted for the number of His^+^ revertants after incubation at 37 °C for 48 h. Control was designed by using distilled water instead of sample. Each experiment was repeated three times. If the colony count of sample-induced revertants is higher than that of the spontaneously occurring revertants, it would suggest that the sample possesses the ability to cause mutagenesis.

### Antimutagenic test

Mutagen 4-nitroquinoline N-oxide (4-NQNO) is a direct mutagen, meaning that it has the ability to cause mutation directly and need not be activated by liver enzyme S9. Mutagen benzo[a]pyrene (B[a]P) is an indirect mutagen, meaning that it requires conversion and activation by the liver enzyme mix S9 to generate the ability of mutation. Mutagen 4-NQNO and B[a]P were prepared in DMSO solution with a concentration of 10 and 50 μg/ml, respectively. The test of antimutagenic ability was performed according to the method described by Maron and Ames [4]. ACE solution was mixed with 0.1 ml mutagen (either 4-NQNO or B[a]P), and 0.5 ml of 0.2 M phosphate buffer (pH 7.4) was added with or without S9 mix. One milliliter of the activated *S. typhimurium* TA98 was then aliquoted into each of the sample tubes. After incubating at 37 °C for 20 min, 2 ml of molten top agar (about 45 °C) was added into each tube, mixed well and then poured into a minimal glucose agar plate. Colonies in the plate were counted for the number of His^+^ revertants after incubation at 37 °C for 48 h.

### Protection effect analysis of DNA damage

Fenton reaction can generate hydroxyl radicals [7]. The radicals would attack the deoxyribose elements of DNA molecules, degrade the molecules by the release of purine and pyrimidine bases, and produce mutagenic sites [8, 9]. By assaying the retention of intact DNA molecules, the protection effect of ACE on DNA damage was evaluated.

Each 45 μl aliquot of a reaction mixture, which was a blend of ACE (0–200 μg total solids/ml), 5 μl of calf thymus DNA solution (25.0 A_260_ unit/ml) (Amersham Biosciences, Piscataway, NJ, USA), 0.9 μl of 3.6 mM FeSO_4_, and 3.6 μl of 24 mM hydrogen peroxide, was incubated at room temperature for 15 min. After incubation, 10 μl of 1 mM EDTA was added to stop the reaction. The blank was the calf thymus DNA solution. The control was the reaction mixture without ACE. Each 10 μl aliquot of the reaction mixture was applied on 1 % agarose gel containing 0.1 % ethidium bromide. The electrophoresis was conducted in TBE buffer (10 mM Tris-boric acid-EDTA, pH 7.4) for 8 min. The gel was then visualized under UV illumination.

## Results and discussion

### Composition of submerged cultured *A. camphorata*

The composition analysis showed that the water content of the dry powder of the submerged cultured *A. camphorata* was 17.55 % (Table 1). In previous studies, the water content was measured to be 68 % in the fresh fruit body of 1 year-old *A. camphorata* and 7.33 % in the dried fruit body of *A. camphorata* [10]. The higher water content in the submerged cultured *A. camphorata* was possibly caused by their higher sugar content, which made it easy to absorb moisture from the humid air. The ash content of the dry powder of submerged cultured *A. camphorata* was measured to be 8.63 %. This result agreed with the content of general mushrooms supposed to have ash contents between 5 and 16 %, which is mainly composed of phosphorus, potassium and other inorganic salt.

**Table 1:** The composition of dried powder of *Antrodia camphorata*

Content^a^				
Components	Moisture (%)^a^	Ash (%)^a^	Crude protein (%)^a^	Total phenolic contents (mg/g extract)^a^
*A. camphorata*	17.55 ± 0.83	8.63 ± 0.33	25.39 ± 2.11	20.00 ± 0.79

^a^Crude extracts were extracted from AC aqueous extract. Each value is expressed as mean ± SD (*n* = 3) Moisture value presented was based on air-dried weight, while the other values presented were based on dry weight

The protein content of dry powder of submerged cultured *A. camphorata* was measured to be 25.39 % (Table 1). The protein content of the *A. camphorata* mycelium and fruit body was measured to be 23.84 and 6.6 %, respectively, in previous studies [10]. This higher protein content should be resulted from the thorough utilization of the nitrogen source in the submerged cultivation.

### Total phenolics content and antioxidant activity of *A. camphorata* extract

It has been reported that phenolic compounds possess antioxidant effects that can be used to clean up the active oxygen and free radicals which can prevent the oxidation of phospholipids in cell membranes and lipids in blood, and that these antioxidant effects can thereby decrease the risk of medical problems due to heart disease or arterial damage, such as strokes. In addition, phenolic compounds have been shown to exhibit anti-mutagenic activity [11, 12]. Analysis showed that the total phenolic content of ACE was estimated to be 20.00 mg/g (Table 1). This implied that the antioxidant activity of ACE should be related with the high content of phenolic compounds.

**Fig. 1:**
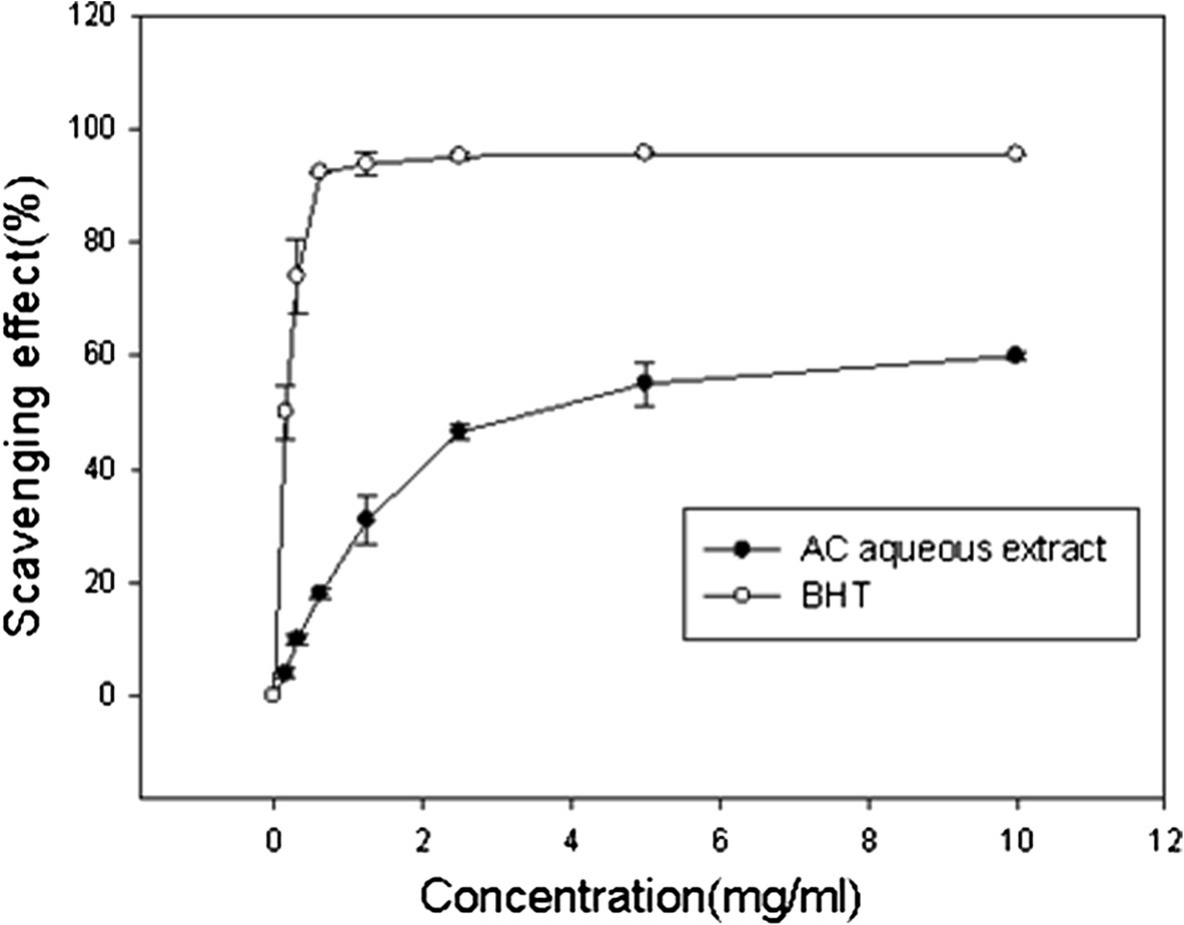
The scavenging effects of *Anthrodia camphorata* extract against hydroxyl radicals. Data are expressed as mean ± SD (*n* = 3). The scavenging effect (%) = [1−(the absorbance of samples at wavelength 517 nm/ the absorbance of control (without sample) at wavelength 517 nm)] × 100 %

Antioxidant agents for inhibiting the lipid oxidation include providing hydrogen to scavenge peroxide radicals, and DPPH is a stable free radical which could accept electrons or hydrogen free radicals to form a stable molecule [13]; therefore, DPPH is a good chemical to generate free radicals and can be used for measuring the antioxidant activity of materials. In the investigation of scavenging ability for DPPH free radicals, results showed that ACE had significant scavenging ability. The scavenging ability was estimated to be 46.53 % when the concentration of ACE was 2.5 mg/ml, and the scavenging ability was proportional to the concentration of ACE. The positive control BHT also showed a scavenging ability of 92.23 % in the concentration of 0.625 mg/ml (Fig. 1). The antioxidant properties of *A. camphorata* were first described by Song et al. in 2002 [14]. It was found DMF and water-extracted ACE showed marked activity in free radical scavengeing and showed that the antioxidant ability of *A. camphorata* is proportional to the total phenolic content [14]; therefore, it is suggested that the scavenging ability of ACE against DPPH radicals should be contributed from the high content of total phenolics. The antioxidant properties of methanolic extracts from *A. camphorata* were also reported [15, 16]. All of studies showed similar results in antioxidant ability, which demonstrated its significant antioxidant activity from the concentration of mg/ml level. Our results agreed with the study reported by Song et al. since they also prepared ACE by water extraction; however, lower antioxidant activity and total phenolics content were observed, it should caused by preparing extract at 4 °C.

### Toxicity and mutagenic ability of *A. camphorata* extract

Before investigating the antimutagenic ability, ACE was used to perform toxicity and mutagenesis tests against *S. typhimurium* TA98, because if the ACE has toxicity and mutagenic effects against *S. typhimurium* TA98, that would result in revertant numbers and an incorrect judgment regarding their antimutagenic effects. According to previous studies, the maintenance of bacteria number after a sample treatment must reach over 80 % of the colony number of the control to prove the sample has no toxic effect [17]. Results of the toxicity test showed that ACE had no significant effect on *S. typhimurium* TA98. (Table 2) However, the bacteria count was proportional to the addition of ACE, which could be reasonably explained by the fact that ACE provides a good nutritional supplement for bacteria growth.

**Table 2:** Toxicity test for *A. camphorata* extract against *S. typhimurum* TA 98 with S9

Dosage (mg/plate)	His^+^ revertants (survival, %)^a^	
	without S9	with S9
0 (control)^b^	1259 ± 6 (100 %)	1355 ± 12 (100 %)
1	1245 ± 10 (99 %)	1338 ± 10 (99 %)
0.5	1255 ± 9 (100 %)	1354 ± 8 (100 %)
0.25	1248 ± 13 (99 %)	1345 ± 6 (99 %)
0.125	1260 ± 12 (100 %)	1363 ± 7 (101 %)
0.0625	1274 ± 7 (101 %)	1365 ± 8 (101 %)

100 μl *S. typhimurium* TA98 was mixed with 100 μl sample and 500 μl phosphate buffer, and incubated at 37 °C for 20 min, after that the population of *S. typhimurium* TA98 was calculated

^a^Each value is expressed as mean ± SD (*n* = 3). Values in parentheses are percentages relative to control value (100 %)

^b^The number of controls was determined without water extracts of *A. camphorata*

According to the judgment criteria in the method, it would be recognized that the sample possessed mutagenic ability when the number of His^+^ revertants induced by the sample was more than twice the number of spontaneous revertants [18]. The mutagenesis test results indicated that ACE had no effect to cause mutagenesis of *S. typhimurium* TA98 whether S9 mix was added or not, since the mutagenicity ratio for ACE against *S. typhimurium* TA98 was between 0.9 and 1.09, indicated that *A. camphorata* extracts induced His^+^ revertants was much less than twice of spontaneous revertants. (Table 3) According to the criteria proposed by Ames et al. in 1975 [18], ACE of experimental concentrations had no toxicity and mutagenic effect. These results were in accordance with the previous study, which showed that DMSO-extracted ACE had no toxic and mutagenic effect [19].

**Table 3:** Mutagenesis test for *A. camphorata* extract against *S. typhimurum* TA 98 with S9

Dosage (mg/plate)	His^+^ revertants (mutagenicity index)^a^	
	without S9	with S9
Spontaneous revertants ^b^	165 ± 10 (1.00)	198 ± 20 (1.00)
1	167 ± 9 (1.01)	186 ± 7 (0.93)
0.5	173 ± 9 (1.04)	197 ± 11 (0.99)
0.25	178 ± 10 (1.07)	202 ± 8 (1.02)
0.125	180 ± 6 (1.09)	203 ± 6 (1.02)
0.0625	173 ± 3 (1.04)	204 ± 12 (1.03)

100 μl *S. typhimurium* TA98 was mixed with 100 μl sample and 500 μl phosphate buffer, and incubated at 37 °C for 20 min, after that the population of *S. typhimurium* TA 98 was calculated

^a^Each value is expressed as mean ± SD (*n* = 3). Mutagenicity index = induced revertants per plate/spontaneous revertants per plate

^b^The number of spontaneous revertants was determined without water extracts of *A. camphorata.* Values in parentheses are mutagenicity index by using spontaneous revertants as 1

### Antimutagenic ability of *A. camphorata* extract

Two mutagens can be used to evaluate the ability of ACE in the antimutagenic test. One is 4-nitro-quinoline-N-oxide (4-NQNO), which need not the activation by liver’s enzyme system. The other one is Benzo[a]pyrene (B[a]P), which requires a liver enzyme to activate its mutagenic ability.

**Fig. 2:**
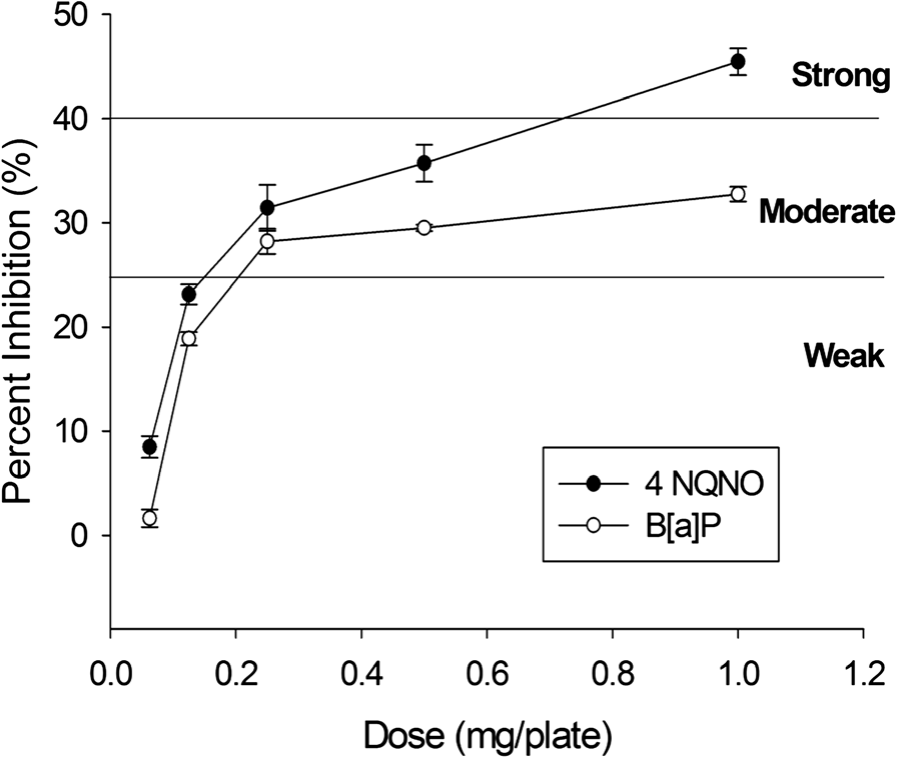
Results of antimutagenic testing against mutagen 4-NQNO and B[a]P by *Anthrodia camphorata* extract. Data are expressed as mean ± SD (*n* = 3). Inhibition rate (%) = [1−number of His^+^ revertants in the presence of *A. camphorata* extract/number of His^+^ revertants in the absence of *A. camphorata* extract] × 100 %

The results of the antimutagenic test showed that ACE has significant effects against 4-NQNO to reduce mutagenesis in different dose treatments with inhibition effects of 7.22, 25.52, 31.82, 36.64, and 44.08 %, and also significant effects against B[a]P in different dose treatments with inhibition effects of 1.85, 18.10, 26.16, 27.45, and 30.05 %. The given antimutagenic effect was in proportion to the concentration of the sample whether 4-NQNO or B[a]P was used as the mutagen generator (Fig. 2). A DMSO-extracted ACE was studied for its mutagenicity in the previous study [19]. Our mutagenic data from water-extracted ACE were consistent with that from DMSO-extracted ACE, which showed no mutagenic effect for *A. camphorata*; however, water-extracted ACE showed better antimutagenic effects on both mutagens than DMSO-extracted ACE. According to the assignment for the inhibition effect of mutagenesis, an inhibitory efficiency higher than 40 % indicates a strong antimutagenic agent; an inhibitory efficiency between 25 and 40 % indicates a moderate antimutagenic agent; and an inhibitory efficiency of lower than 25 % indicates a weak antimutagenic agent [7]. Therefore, ACE was suggested to be a strong antimutagenic agent against mutagen 4-NQNO and a moderate antimutagenic agent against mutagen B[a]P. It is the first report to address water-extracted ACE to be a strong or moderate antimutagenic agent against both mutagens.

### *A. camphorata* extract decreases the DNA damage induced by hydroxyl radicals

**Fig. 3:**
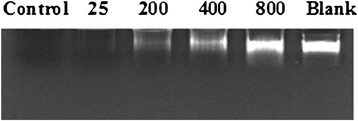
Results of a calf thymus DNA protection test against hydroxyl radical-induced damage by *Anthrodia camphorata* extract. Samples were a blend of *A. camphorata* extract (μg total solids/ml), calf thymus DNA solution, FeSO_4_, and hydrogen peroxide; Blank was calf thymus DNA solution; Control was the reaction mixture containing no *A. camphorata* extract

Oxidative damage to DNA is one of the most important mechanisms in the initiation of cancer. Such damage is usually caused by hydroxyl radicals [20]. The activity of these radicals can be reduced by natural antioxidants found in plants including many herbs [21]. The Fenton reaction involves the reaction between hydrogen peroxide and Fe^2+^ to form hydroxyl radicals. Scavengers of hydroxyl radicals inhibit this reaction through the reduction of Fe^2+^ [8, 9]. It was found that ACE at a concentration of 0.8 mg/ml was able to reduce the hydroxyl radical-induced damage in calf thymus DNA by approximately 82.8 % (Fig. 3). Since ACE possesses good antioxidant activity, its protective effects on DNA should have contribution from its antioxidant activity.

## Conclusions

*A. camphorata* extract (ACE) was found to be a good antioxidant by its good scavenging ability against DPPH radicals. ACE showed no toxicity and mutagenic effect in the toxicity and mutagenic ability studies; moreover, it demonstrated good anti-mutagenic ability against both direct mutagen 4-NQNO and indirect mutagen B[a]P. ACE showed the DNA-protective activity by its good ability in reducing hydroxyl radical-induced DNA damage. These results suggested that *A. camphorata* is a new and novel material for health foods with antioxidant, antimutagenic and DNA-protective activities.

## Abbreviations

ACE: *Antrodia camphorata* extract
DPPH: 2,2-diphenyl-1-picrylhydrozyl
4NQNO: 4-nitroquinoline N-oxide
B[a]P: benzo[a]pyrene
BHT: butylated hydroxytoluene
DMSO: dimethyl sulfoxide
